# Ultrafine NaTi_2_(PO_4_)_3_ Nanoparticles Encapsulated in N-CNFs as Ultra-Stable Electrode for Sodium Storage

**DOI:** 10.3389/fchem.2018.00270

**Published:** 2018-07-06

**Authors:** Sicen Yu, Yi Wan, Chaoqun Shang, Zhenyu Wang, Liangjun Zhou, Jianli Zou, Hua Cheng, Zhouguang Lu

**Affiliations:** Department of Materials Science and Engineering, Southern University of Science and Technology, Shenzhen, China

**Keywords:** NTP, N-doping carbon nanofibers, cycling performance, electrospinning, sodium-storage

## Abstract

We present a feasible method for the preparation of one-dimensional N-doping carbon nanofibers encapsulated NaTi_2_(PO_4_)_3_ (NTP-NCNFs) through electrospinning accompanied by calcination. The poor electrical conductivity of NTP is significantly improved and the as-prepared NTP-NCNFs exhibit stable and ultrafast sodium-storage capability. The NTP-NCNFs maintains a stable specific capacity of 121 mAh g^−1^ at 10 C after 2,000 cycles, which only drop to 105 mAh g^−1^ after 20,000 cycles. Furthermore, the NTP-NCNFs show excellent rate performance from 0.2 to 20 C, whose recovery efficiency still reaches 99.43%. The superior electrochemical property is mainly attributed to the large specific surface area, high porosity, N-doping carbon coating, and one-dimensional structure of NTP-NCNFs.

## Introduction

Because of the high energy density, stable cycling performance, and environmental benignity, Li-ion batteries have been widely applied in portable devices and electric vehicles (Duncan et al., 2016; Kim et al., 2016; Zhang et al., 2018; Zheng et al., 2018). However, the limited lithium mineral reserves restrict the wide application of LIBs in grid-scale energy storage system. As cost-effective alternatives to Li-ion batteries, sodium ion batteries have been investigated for next-generation energy storage system, benefiting from sodium abundance (Wu et al., 2016; Wang et al., 2017, 2018; Xu et al., 2017). SIBs basically have similar battery components and electrical storage mechanisms as LIBs. However, the poor electrochemical kinetics and large volume change caused by large size of Na ions leads to severe capacity loss and cycling degradation. Therefore, the challenge of looking for sodium storage materials with good stability and high-rate capacity still remains.

Recently, NTP as one of sodium super-ionic conductor (NASICON) has been demonstrated as potential long life-time and high-rate electrode material for SIBs (Guin and Tietz, 2015; Wu et al., 2015; Hu et al., 2018; Liang L. et al., 2018). The strong P-O covalent bond in the phosphates offers remarkable structural and thermal stability and the open three-dimensional (3D) framework in NTP allows for fast sodium ions transfer(Li et al., 2012; Yang et al., 2015; Wang et al., 2016). However, pristine NTP with low intrinsic electronic conductivity displays poor electrochemical performance. In order to address this issue, some strategies including nano-sizing the particle, coating a conductive layer on the surface, and mixing with high conductive materials have been proposed (Fang et al., 2016; Ha-Kyung et al., 2016; Liang et al., 2018b). Although significant enhancement has been achieved, the satisfactory electrochemical performance with high rate capability and stability of NTP is still of great urgent.

Electrospinning is a fascinating way to prepare CNFs (Li et al., 2017; Zhu et al., 2017; Liang et al., 2018a). Meanwhile, due to the presence of carbon and nitrogen source from the starting polymer, electrospinning can be adapted to a feasible preparation for N-doping carbon matrixes encapsulated NTP nanoparticles to realize high performance for sodium storage. Herein, NTP nanoparticles are embeded into conductive N-doping carbon nanofibers (denoted as NTP-NCNFs). 1D nanofibers provide fast charge transfer pathway, ensuring the NTP-NCNFs with superb rate performance. The NCNFs matrix also contributes to the ultra-long cycling stability. At 10 C rate, the NTP-NCNFs maintained a specific capacity of 105 mAh g^−1^ after 20,000 cycles.

## Experimental section

### Preparation of NTP-NCNFs

NaH_2_PO_4_·2H_2_O (0.211 g) and polyacrylonitrile (PAN) (0.8 g) were dissolved into N, N-dimethylformamide (DMF) (10 ml) to obtain a homogeneous solution. Then, titanium tetraisopropanolate [(CH_3_CH_3_CHO)_3_Ti] (0.5 ml) was dropped into above solution and stirred overnight. And then, the solution was injected into the syringe with a 21 G needle, which the flow rate was 10 uL min^−1^. Al foil was employed as the collector with distance to the needle of 15 cm and voltage of 15 kV. The as-electrospun fibers were carbonized in a tube furnace at 700°C (denoted as 700NTP-NCNFs), 800°C (denoted as 800NTP-NCNFs), 900°C (TiN) for 2 h under inert atmosphere. As a fair comparison, the NaTi_2_(PO_4_)_3_ powder was synthesized via mixing the same ratio of NaH_2_PO_4_·2H_2_O and [(CH_3_CH_3_CHO)_3_Ti] annealed at 800°C under argon atmosphere.

### Materials characterization

The crystal structure of the fibers was characterized using powder X-ray diffraction (XRD) on a Rigaku D/Max-2400 X-ray diffractometer with Cu-Kα radiation (λ = 1.54056 Å). The specific surface area and the pore size distribution of as-prepared N-doping carbon coating NTP nanofibers (700 and 800°C) were evaluated by the Brunauer-Emmet-Teller (BET) at 77 K using a NOVA 1200e Surface Area. Raman spectra of samples were acquired with a Lab RAM HR 800 Raman microscope with an excitation laser beam (λ = 532 nm). SEM images were obtained on a scanning electron microscope (Hitachi, S4800) attached with an energy-dispersive X-ray spectroscopy (EDS) facility. TEM and HR-TEM images were recorded on a JEOL JEM-2010 (JEOL Ltd, Tokyo, Japan) at 200 kV. *Ex-situ* XPS records were performed on a VG scientific ESCALAB 250 spectrometer.

### Electrochemical analysis

The electrochemical properties of the N-doping carbon coating NTP nanofibers was tested by assembling 2016 coin-type cells with Na as the counter electrode. NTP-NCNFs, Super P, and polyvinylidene fluoride (PVDF) binders dissolved in N-methylpyrrolidone (NMP) were mixed into slurry with a weight ratio of 8:1:1, which was coated on a Cu foil and dried in a vacuum oven at 110°C for 6 h, further dividing into wafers with 12 mm diameter. The active material is about 0.64 mg cm^−2^. The separator was a glass fiber filter. The electrolyte was 1 M NaClO_4_/ethylene carbonate (EC) and propylene carbonate (PC) with volume ratio of 1:1. The specific capacity is based on the whole mass of NTP-NCNFs. Electrochemical capacity measurements of the NTP-NCNFs were tested on the Neware battery test system by applying galvanostatic charge-discharge. Cyclic voltammetry (CV) was performed on a BioLogic-VMP3 electrochemical workstation at the same voltage window with a sweep rate of 0.1 mV s^−1^. Electrochemical impedance spectroscopy (EIS) was recorded at an AC voltage of 5 mV amplitude in the frequency range from 1.0 to 100 mHz at room temperature.

## Results and discussion

**Figure 1:**
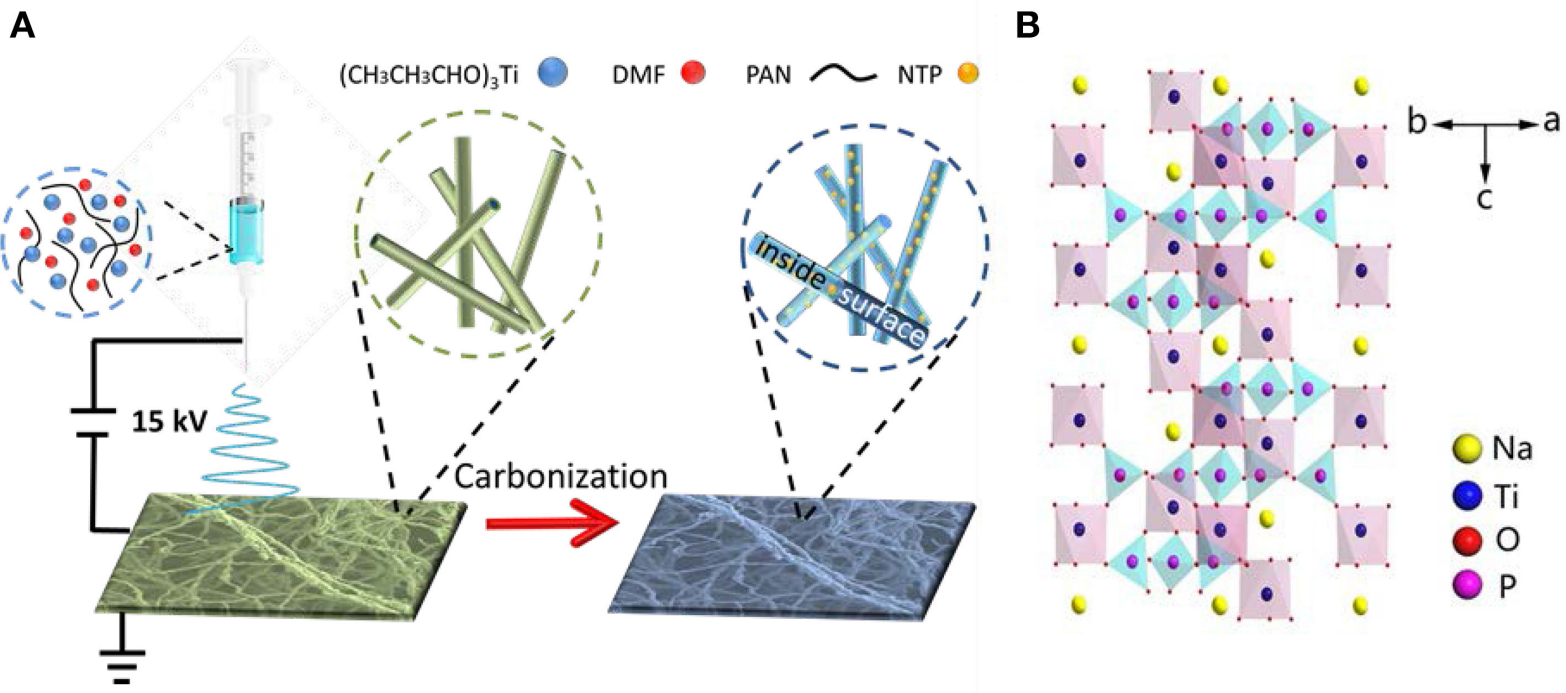
Schematic representation of **(A)** the synthesis process and **(B)** the structure illustration of NTP-NCNFs.

**Figure 2:**
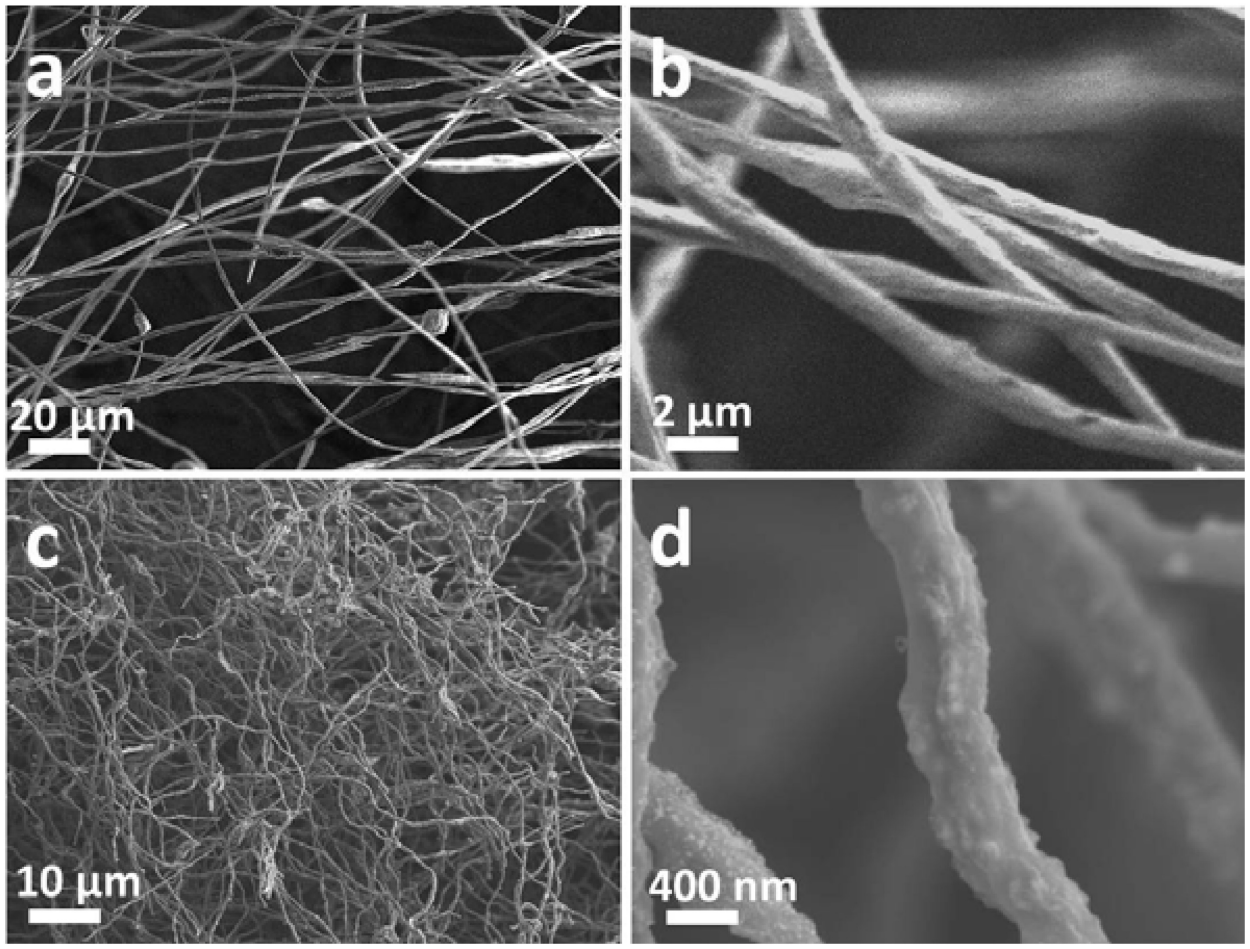
SEM images of 800NTP-NCNFs before **(a,b)** and after **(c,d)** calcination.

Figure 1 depicts the synthesis of NTP-NCNFs. It should be noted that (CH_3_CH_3_CHO)_3_Ti was added into the mixture solution under vigorous stirring followed by electrospinning. Figures 2a,b

show the continuous nanofibers with diameter of ~ 500 nm. The as-prepared nanofibers will be subjected to a calcination process under argon atmosphere at 800°C, during which the PAN will convert into N-doped carbon materials while keeping its 1D morphology (Wang et al., 2013). The result of thermogravimetric analysis shows that the mass ratio between NaTi_2_(PO_4_)_3_ and N-doped carbon materials is about 1:1 (Figure S1). Meanwhile, the NaH_2_PO_4_ and (CH_3_CH_3_CHO)_3_Ti form NTP nanoparticles via a solid reaction process (Ribero et al., 2016; Wang et al., 2016). As shown in Figures 2c,d, the surface of the fiber become rough and evenly decorated by nanoparticles after calcination. However, after pyrolysis at 900°C in argon atmosphere, the as-prepared sample has changed to TiN (JCPDF#65-0715) as demonstrated by the XRD analysis (Figure S2). This might be caused by the reduction of NTP at higher pyrolysis temperature by ammonia, which was formed by the decomposition of PAN (Liu et al., 2013). As-prepared TiN still keeps as nanofibers, shown in Figure S3. Though it is beneficial to the electrical conductivity of NTP-NCNFs, the further pyrolysis of carbon layer leads to a bad influence on its electrochemical performance, which was exhibited in Figure S4. Therefore, the further research mainly focuses on 700/800NTP-NCNFs.

**Figure 3:**
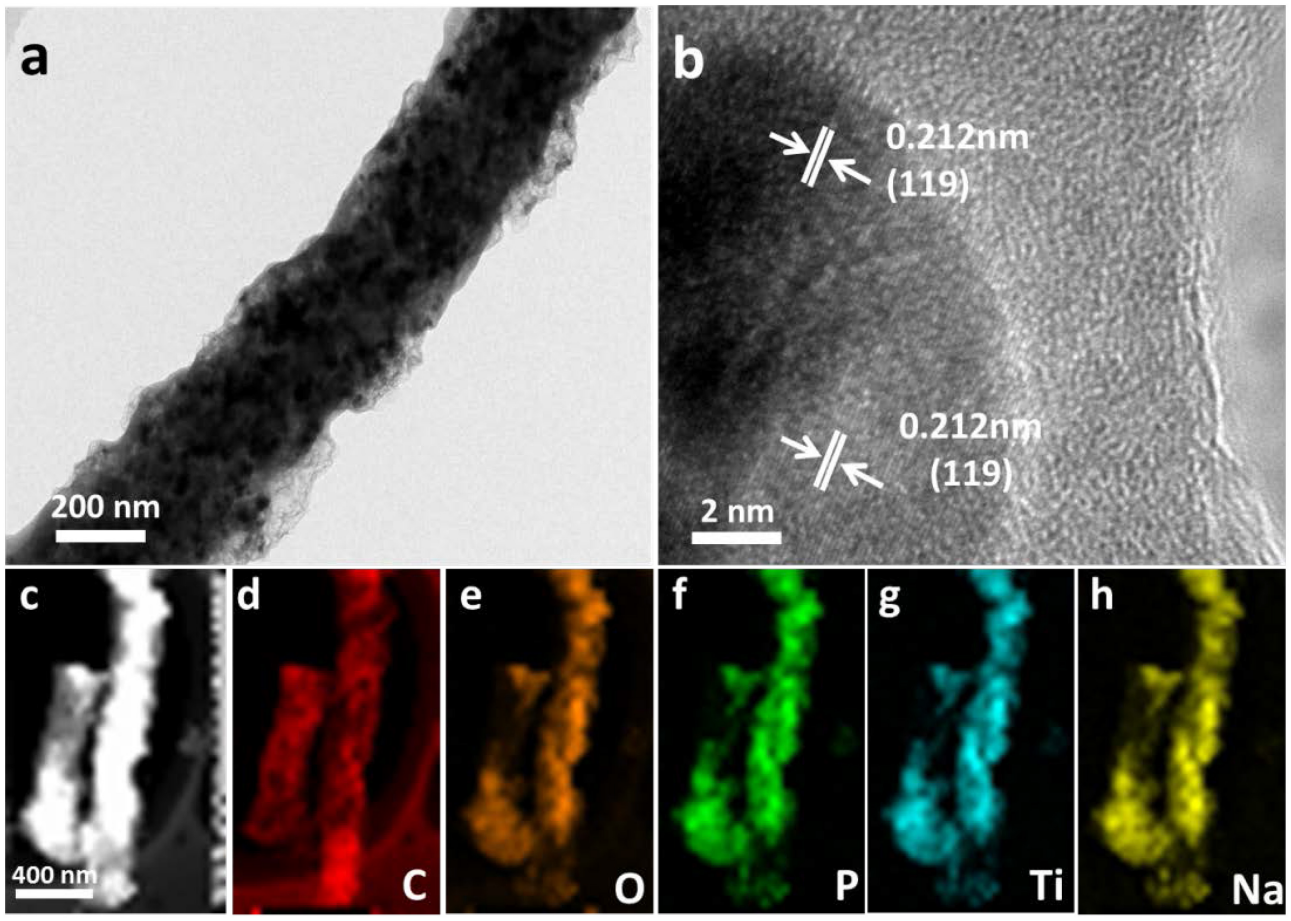
**(a)** Typical TEM image and **(b)** HRTEM image of 800NTP-NCNFs, **(c)** HAADF/STEM image of 800NTP-NCNFs, and corresponding EDS elemental mapping images: **(d)** C, **(e)** O, **(f)** P, **(g)** Ti, and **(h)** Na.

High resolution TEM (HRTEM) measurements further confirmed the formation of NTP. Figure 3a shows that NTP nanoparticles evenly distributed over the carbon fiber. HRTEM (Figure 3b) reveals the lattice fringes of 0.21 nm, matching with the spacing of (119) plane of NTP. The elemental mapping using EDS coupled with HAADF/STEM was utilized to investigate the compositional distributions of C, O, P, Ti, and Na in 800NTP-NCNFs (Figures 3c–h), which clearly shows that these elements are uniformly distributed in 800NTP-NCNFs. The uniform structure of N-doping carbon matrix should improve the electrical conductivity of NTP.

**Figure 4:**
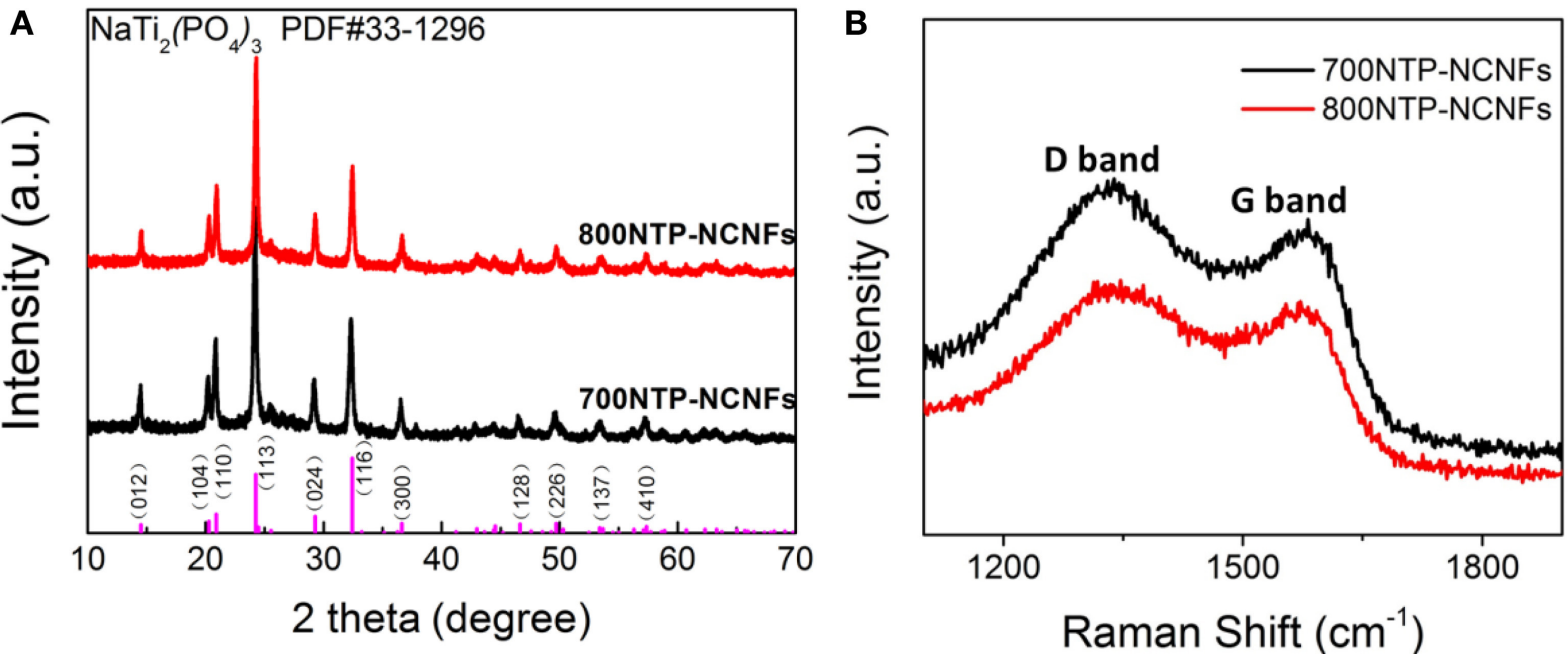
**(A)** The X-ray diffraction patterns and **(B)** Raman spectrum of 700NTP-NCNFs and 800NTP-NCNFs.

Figure 4A shows the XRD patterns of NTP-NCNFs, in which all the peaks can be indexed to the standard NTP peaks (JCPDF#33-1296). To clarify the degrees of defect structure and graphitization, Raman spectroscopy is carried out. As shown in Figure 4B, the D (1,350 cm^−1^) and G (1,600 cm^−1^) bands are significantly observed, which represents disordered and graphitization, respectively. The I_D_/I_G_ value of 800NTP-NCNFs is 1.05, which is evidently lower than that of 700NTP-NCNFs (1.14) (Wang et al., 2015). The significantly decreased value of I_D_/I_G_ indicates an enhanced degree of graphitization, which is likely beneficial to fast electron transport of NTP-NCNFs.

**Figure 5:**
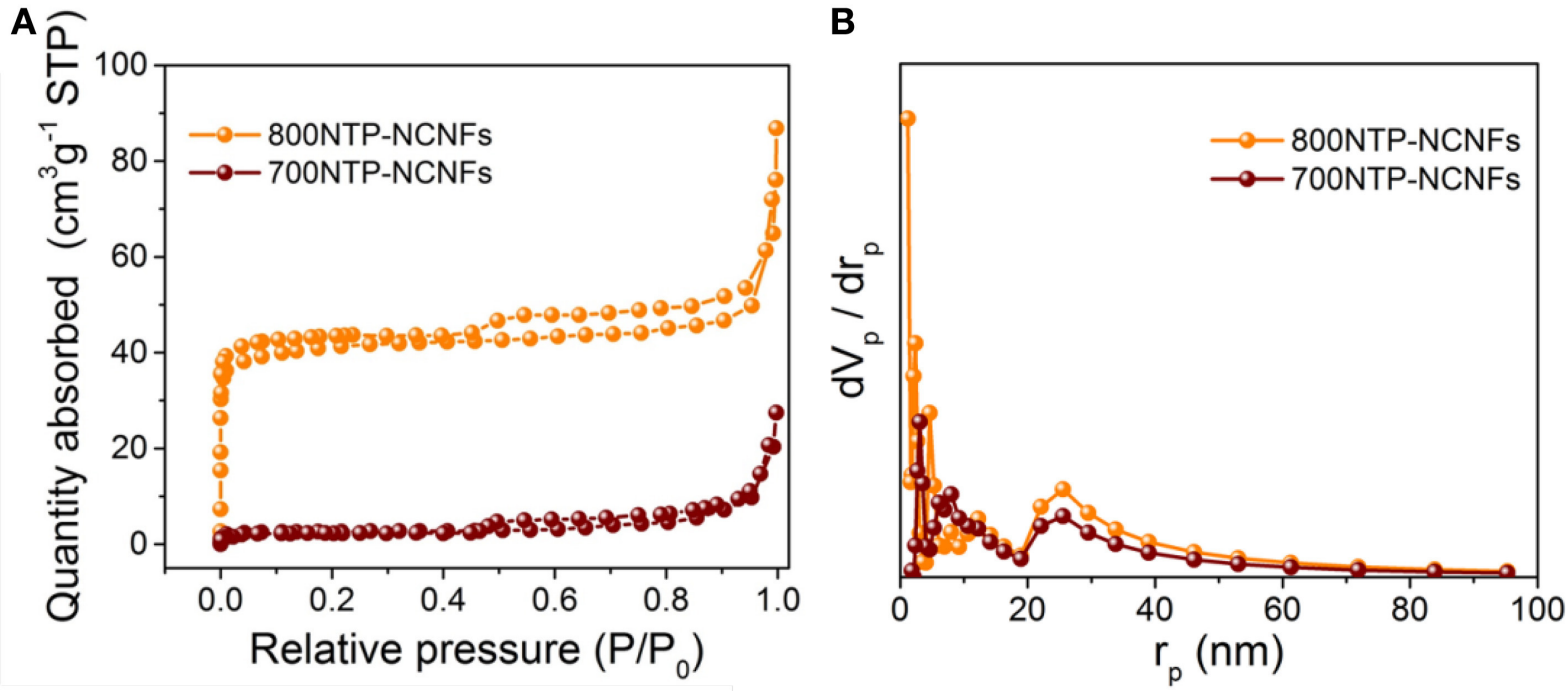
**(A)** N_2_ absorption-desorption isotherm and **(B)** the pore-size distribution curves of the 700NTP-NCNFs and 800NTP-NCNFs.

N_2_ adsorption and desorption isotherms (Figure 5A) of both 700NTP-NCNFs and 800NTP-NCNFs can be identified as type IV isotherms, with pronounced hysteresis loops, implying the mesopores in NTP-NCNFs. The BET surface area of 800NTP-NCNFs is 153.86 m^2^ g^−1^, which is much higher than that of 700NTP-NCNFs (~10 m^2^ g^−1^). The high BET surface area of 800NTP-NCNFs is likely due to the higher carbonization degree and the release of gas generated at higher pyrolysis temperature. The pore size distributions are shown in Figure 5B, which suggest the formation of micro-, meso-, and macro-pores. The larger specific surface area and hierarchical pore structures can effectively increase the mass transport and the contact between NTP and electrolyte, which would be beneficial to the electrochemical performance of the 800NTP-NCNFs. Therefore, we believe that 800NTP-NCNFs will have better electrochemical performance than 700NTP-NCNFs (Figure S5).

**Figure 6:**
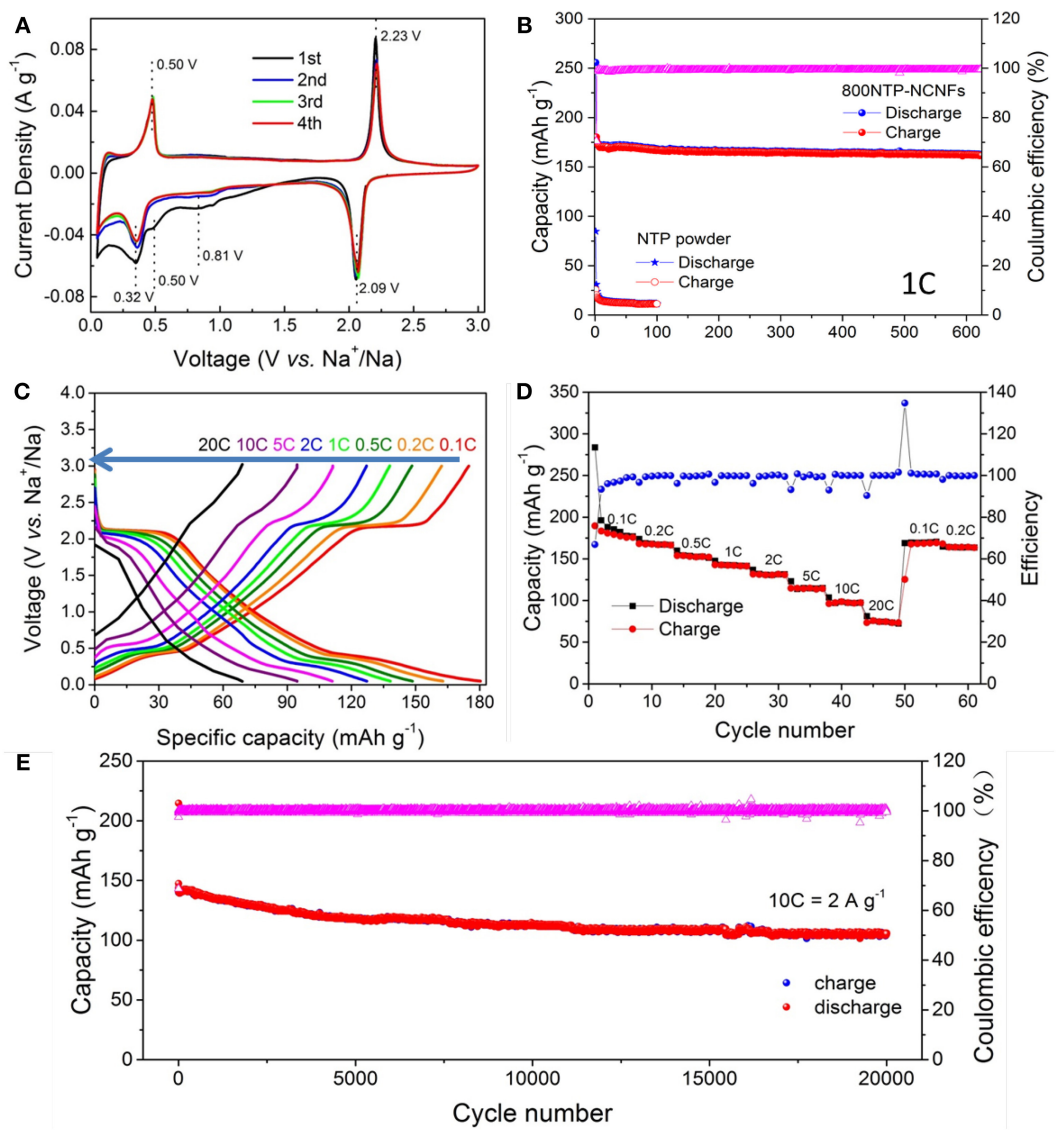
Electrochemical performance of 800NTP-NCNFs: **(A)** CV curves for the initial 4 cycles, **(B)** the cycling performance at a current density of 200 mA g^−1^ (1 C) and corresponding coulombic efficiency, **(C)** galvanostatic discharge-charge profiles at different current rates with initial discharge capacity excluded, **(D)** rate capability at different current rates ranging from 0.1 to 20 C and back to 0.1–0.2 C, **(E)** cycle performance at high current density of 2 A g^−1^ (10 C).

We first use cyclic voltammetry (CV) to investigate the sodium storage mechanism of 800NTP-NCNFs (Figure 6A). During the initial cathodic scan, the peaks at 0.81 and 0.51 V are higher than those of subsequent cycles, which are caused by the formation of solid electrolyte interface layer (SEI). Remarkably, nearly all the shape and the position of the peaks are overlap after the second CV curve, suggesting excellent electrochemical reversibility and stability of 800NTP-NCNFs for sodium storage.

The discharge-charge profiles of 800NTP-NCNFs are recorded at various rates (Figure 6C). The plateaus at ~2.1 and ~0.32 V during discharge and plateaus at ~0.5 and ~2.2 V during charge are in agreement with the CV results. Here, the NCNFs contribute to Na^+^ storage and the overall specific capacity is based on the whole mass of 800NTP-NCNFs (Stevens and Dahna, 2000; Dahbi et al., 2014).

Figure 6D shows the rate performance of 800NTP-NCNFs at different current densities. The reversible specific capacity was 176 mAh g^−1^ at 0.1 C. As the rate increased from 0.2 to 0.5, 1, 2, 5, 10, and 20 C, the corresponding specific capacity was 163, 149, 138, 127, 110, 95, and 71 mAh g^−1^, with a capacity retention ration of 92.61, 84.66, 78.41, 72.16, 62.50, 53.98, and 40.34% as reference to that at 0.1 C, respectively. As the rate returned to 0.1 C, the specific capacity of 800NTP-NCNFs recovered to 175 mAh g^−1^, which demonstrated the excellent reversibility of 800NTP-NCNFs during Na^+^ insertion and extraction. As illustrated in Figure 6E, the 800NTP-NCNFs deliver ultra-stable cycling performance and rate capability for 20,000 cycles at 10 C. The 800NTP-NCNFs maintain a stable specific capacity of 121 mAh g^−1^ after 2,000 cycles, which is about 80% of initial capacity. After 20,000 cycles, the 800NTP-NCNFs even deliver a considerable capacity of 105 mAh g^−1^. The astonishing electrochemical property of 800NTP-NCNFs is attributed to several features: (i) NASICON-structured NTP ensures sufficient ion transport; (ii) N-doping carbon coating on the surface of NTP improves the electronic conductivity of NTP, which provides fast electronic transport; (iii) 1D structure with large surface area ensures rapid ion transport by enhancing electrode and electrolyte contact; (iv) the hierarchical pore distribution is favorable for the electrolyte penetration and accommodation of volume change.

**Figure 7:**
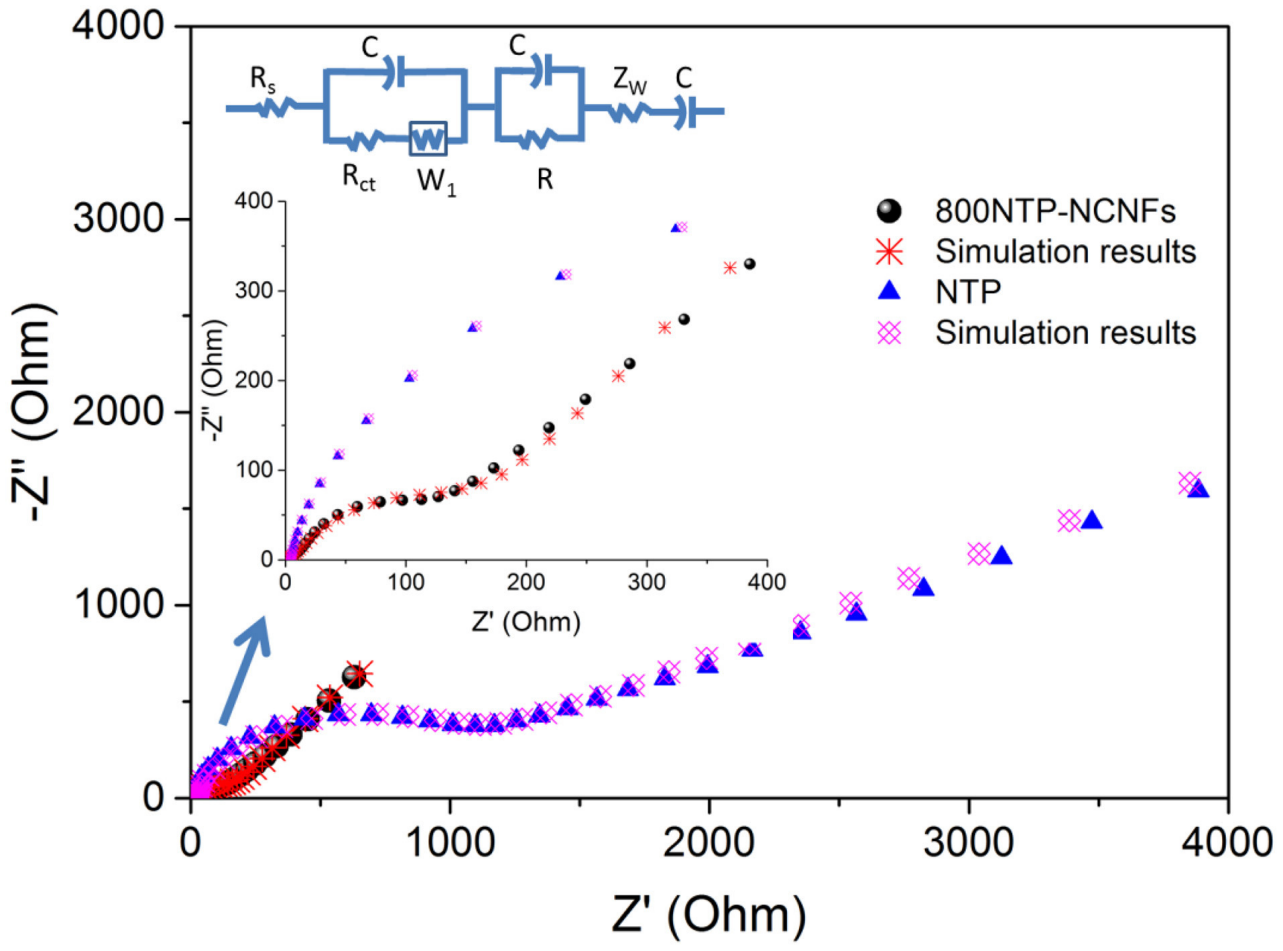
Electrochemical impedance spectroscopy (EIS) measurements of 800NTP-NCNFs and pristine NTP powders.

The 800NTP-NCNFs displayed superior cycling stability with high specific capacity to that of pristine NTP powder (Figure 6B), which further demonstrated that 800NTP-NCNFs intrinsically improved the electronic conductivity of NTP. To investigate the improvement of 800NTP-NCNFs, EIS was performed in Figure 7. The charge-transfer resistance (R_ct_) for pristine NTP powder electrode is 1100 Ω, and this high resistance reflects the low intrinsic electrical conductivity of NTP. The R_ct_ of 800NTP-NCNFs is significantly lower than that of pristine NTP powder electrode (around 150 Ω), which implies that NTP encapsulated in N-doping carbon fiber structure successfully enhance the electrical conductivity and, in turn, the electrochemical performance of NTP (Figure 6B).

## Conclusion

In summary, we presented a feasible method to prepare NTP-NCNFs which exhibits excellent rate capability and stable cycling performance for sodium storage. The NTP-NCNFs could deliver a specific capacity of 105 mAh g^−1^ under 10 C even after 20000 cycles. The nanosized NTP shorten the solid-state ion diffusion length and accelerate surface electrochemical reaction in the electrode. The 1D N-doped carbon coating enhances electronic conductivity of NTP and ensures the fast electron transfer. Moreover, the 3D woven network is beneficial to the penetration of electrolyte and accommodates the volume change of NTP during cycling.

## Associated content

### Supporting information

TGA curve of 800NTP-NCNFs, XRD patterns of TiN, SEM and TEM images of TiN, and electrochemical performance of TiN and 700 NTP-NCNFs including CV curves for the initial 4 cycles and the cycling performance at a current density of 200 mA g^−1^ (1 C) and corresponding coulombic efficiency.

## Author contributions

ZL and CS designed the research. CS, SY, YW, ZW, and LZ performed the experiments. JZ, HC, ZL, and CS incorporated in the interpretation of experimental results. All authors participated in the general discussion.

### Conflict of interest statement

The authors declare that the research was conducted in the absence of any commercial or financial relationships that could be construed as a potential conflict of interest.
