# Supplementary material for: Ultrafine NaTi2(PO4)3 Nanoparticles Encapsulated in N-CNFs as Ultra-Stable Electrode for Sodium Storage
Source: Front Chem. 2018 Jul 6;6:270. doi: 10.3389/fchem.2018.00270 (PMC6043649; doi:10.3389/fchem.2018.00270)
Supplement: Supplementary file 1 [file Data_Sheet_1.DOCX]

Supporting Information

Ultrafine NaTi_2_(PO_4_)_3_ Nanoparticles Encapsulated in N-CNFs as Ultra-stable Electrode for Sodium Storage

Sicen Yu, Yi Wan, Chaoqun Shang, Zhenyu Wang, Liangjun Zhou, Jianli Zou, Hua Cheng*, Zhouguang Lu*

Department of Materials Science and Engineering, Southern University of Science and Technology, Shenzhen, China

*Corresponding author.

Tel: (+86) 755-88018966

E-mail: [luzg@sustc.edu.cn](mailto:luzg@sustc.edu.cn), chengh@sustc.edu.cn

Supporting figures:


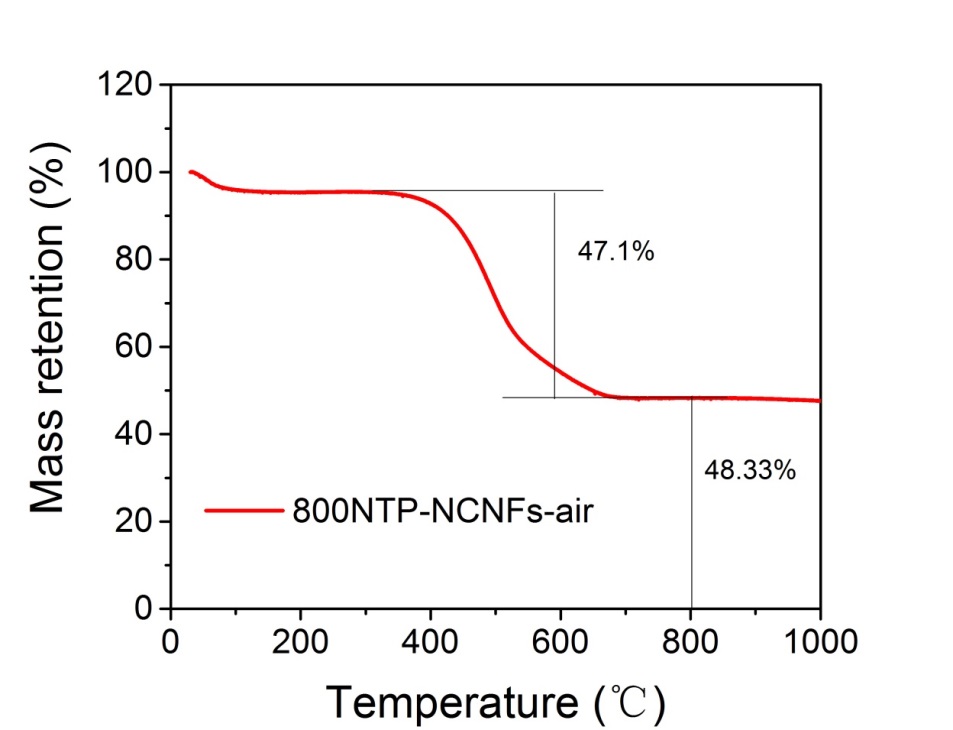


Figure S1. TGA curve of 800NTP-NCNFs.


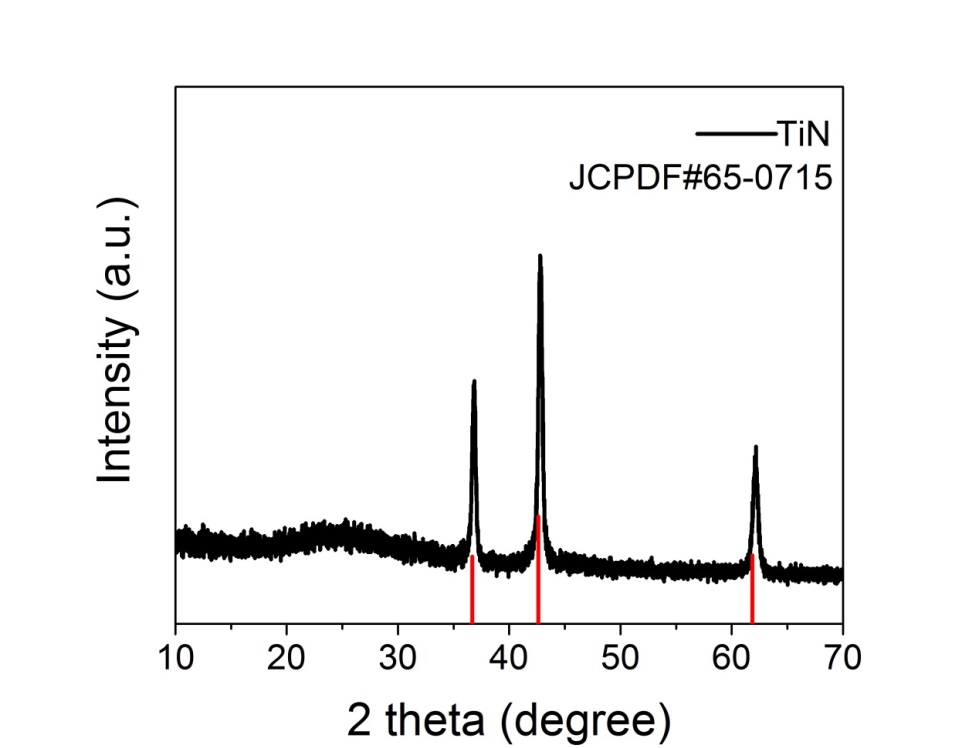


Figure S2. XRD pattern of TiN.


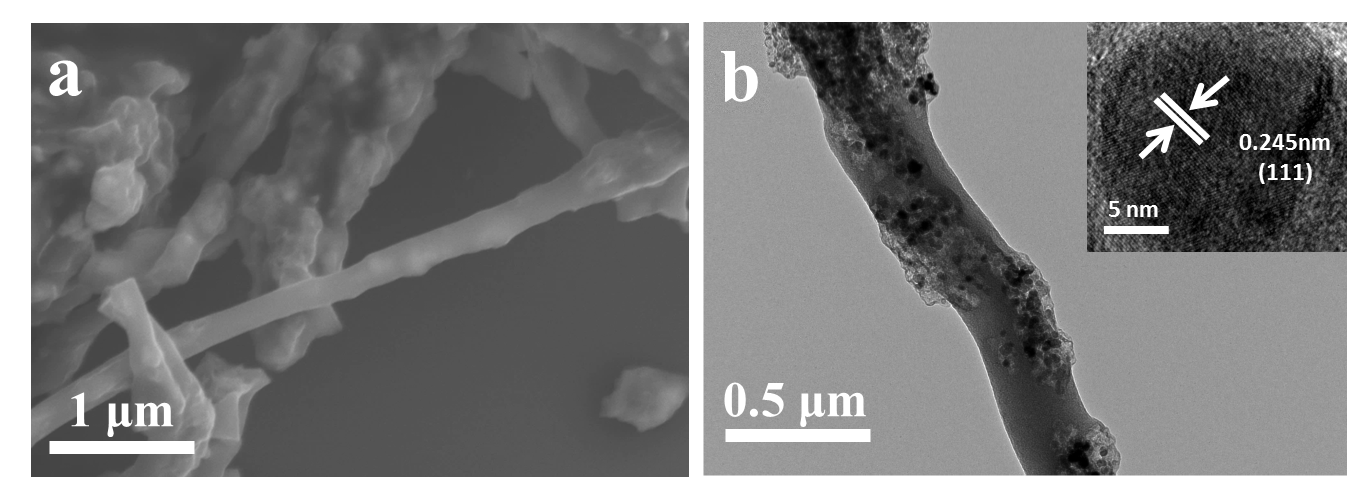


Figure S3. (a)SEM and (b)TEM images of TiN.


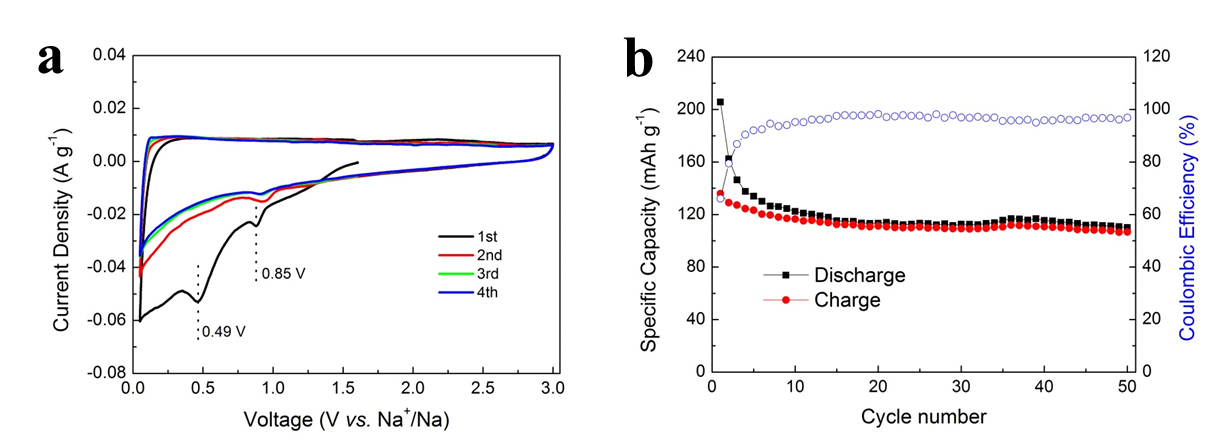


Figure S4. Electrochemical performance of TiN: (a) CV curves for the initial 4 cycles, (b) the cycling performance at a current density of 200 mA g^-1^ (1 C) and corresponding coulombic efficiency.


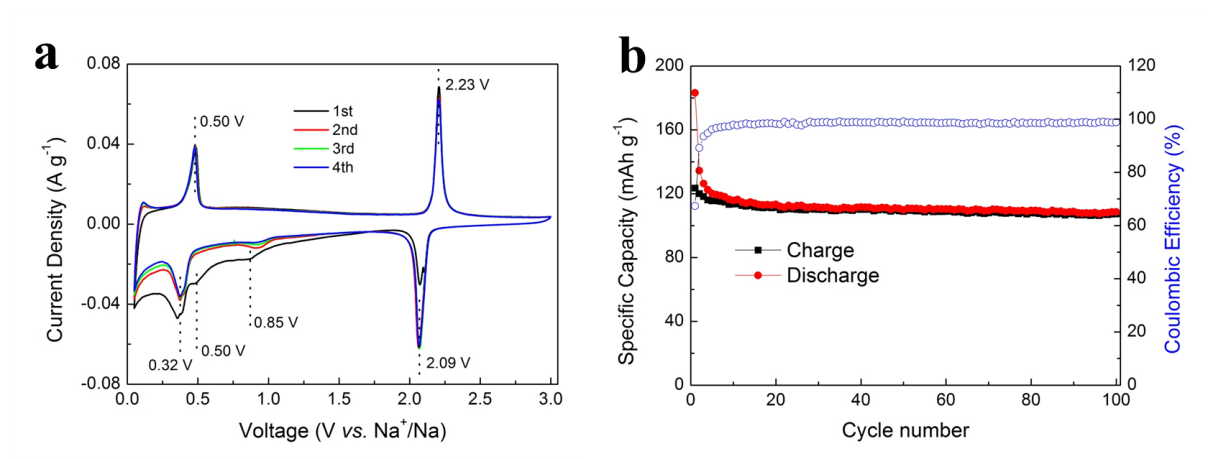


Figure S5. Electrochemical performance of 700 NTP-NCNFs: (a) CV curves for the initial 4 cycles, (b) the cycling performance at a current density of 200 mA g^-1^ (1 C) and corresponding coulombic efficiency.
